# A new protective gel to facilitate ulcer healing in artificial ulcers following oesophageal endoscopic submucosal dissection: a multicentre, randomized trial

**DOI:** 10.1038/s41598-023-33982-7

**Published:** 2023-04-26

**Authors:** Tianyu Zhou, Xinli Mao, Lei Xu, Haifeng Jin, Li Cen, Caijuan Dong, Linying Xin, Jiali Wu, Weimiao Lin, Bin Lv, Feng Ji, Chaohui Yu, Zhe Shen

**Affiliations:** 1 Department of Gastroenterology, Zhejiang University School of Medicine First Affiliated Hospital, Hangzhou, 310003 Zhejiang China; 2 Department of Gastroenterology, Taizhou Hospital of Zhejiang Province, Taizhou, Zhejiang China; 3 Department of Gastroenterology, Ningbo City First Hospital, Ningbo, Zhejiang China; 4 Department of Gastroenterology, Zhejiang Provincial Hospital of Chinese Medicine, Hangzhou, Zhejiang China

**Keywords:** Gastrointestinal system, Oesophagogastroscopy

## Abstract

There are significant risks of adverse events following oesophageal endoscopic submucosal dissection (ESD), such as stricture, delayed bleeding and perforation. Therefore, it is necessary to protect artificial ulcers and promote the healing process. The current study was performed to investigate the protective role of a novel gel against oesophageal ESD-associated wounds. This was a multicentre, randomized, single-blind, controlled trial that recruited participants who underwent oesophageal ESD in four hospitals in China. Participants were randomly assigned to the control or experimental group in a 1:1 ratio and the gel was used after ESD in the latter. Masking of the study group allocations was only attempted for participants. The participants were instructed to report any adverse events on post-ESD days 1, 14, and 30. Moreover, repeat endoscopy was performed at the 2-week follow-up to confirm wound healing. Among the 92 recruited patients, 81 completed the study. In the experimental group, the healing rates were significantly higher than those in the control group (83.89 ± 9.51% vs. 73.28 ± 17.81%, *P* = 0.0013). Participants reported no severe adverse events during the follow-up period. In conclusion, this novel gel could safely, effectively, and conveniently accelerate wound healing following oesophageal ESD. Therefore, we recommend applying this gel in daily clinical practice.

## Introduction

Endoscopic submucosal dissection (ESD) is a popular intervention for digestive system neoplasms. Moreover, ESD is used to perform en bloc resection of entire oesophageal neoplasms, contributing to a precise pathological assessment^1,2^. The common adverse events related to oesophageal ESD include delayed bleeding, perforation, and stricture^3^. Among them, stenosis is the main long-term complication of oesophageal ESD, which occurs in 10–20% of patients undergoing ESD in the oesophagus^3^. Furthermore, the risk of oesophageal stricture can increase up to 90% when ESD exceeds three-quarters of the oesophageal circumference^4–6^. Oesophageal stricture can result in dysphagia, vomiting, weight loss, and a substantially reduced quality of life. To date, the circumferential extension of the artificial ulcer, longitudinal length of the mucosal defect, and histological depth have been reported to contribute to oesophageal stricture after ESD^7–9^.

Currently, endoscopic balloon dilatation, stent placement, oral steroid administration, and endoscopic steroid injection have been used prophylactically for strictures after ESD^10^. Stricture prevention and resolution could impair oesophageal motility, which may further cause dysphagia^11,12^. Additionally, corticosteroid administration can cause severe infectious morbidity with suboptimal efficacy^13–15^. The exposure of the submucosal space to microbiological factors and chemical factors induces an inflammatory and fibrogenic reaction and can lead to oesophageal stricture^15,16^. The ulcer-healing process starts with local recruitment of inflammatory cells and fibroblasts, at the time when fibrous hyperplasia is evident^17^. Moreover, it was reported that larger mucosal defects are significantly associated with oesophageal stricture after ESD^2^. Therefore, it is necessary to protect artificial ulcers and promote the healing process to prevent oesophageal stricture after ESD. Apart from conventional techniques, advanced strategies such as stimulating epithelial growth with growth factors, cell sheets, or covering the wound with mineral or organic sheets have been proposed to protect wounds after ESD^18–21^. Tissue engineering and autologous tissue transplantation are potential preventive methods and are currently used in animal experiments. However, tissue engineering and autologous tissue transplantation require high technical surgeon expertise with a long fixation time^22,23^. A polyglycolic acid (PGA) sheet can promote cell migration during the healing process, thereby reducing the risk of oesophageal stricture. Nevertheless, the delivery and fixation of PGA sheets are time-consuming, and PGA sheets can easily be disturbed by food^24^. It is necessary to develop new strategies to address these concerns.

We designed a novel gel consisting of a colloidal and fixative solution, to protect artificial wounds after oesophageal ESD. The colloidal solution is composed of poloxamers and sodium alginate, whereas the fixative solution contains calcium chloride. Poloxamers are synthetic nonionic triblock copolymers (PEO–PPO–PEO) of polyethylene oxide, and the most notable features of poloxamers include their temperature-dependent self-assembly and thermo-reversibility^25,26^. The hydrogels maintain a fluid state at room temperature, but become a semisolid gel at body temperature, and when the temperature decreases, they return to the fluid state^25^. Poloxamers demonstrate strong water solubility, low biotoxicity, low stimulation for organisms, and versatile biocompatibility. These features have allowed poloxamers to be used in forming micro/nanofibers, cell carrier constructs, and drug micro/nanocarriers in diverse applications^26^. Recently, due to their versatility, biomaterials composed of poloxamers have been used in multiple areas of tissue engineering, such as neurogenesis, angiogenesis, and bone regeneration^26^. Sodium alginate is a natural polymer that is nontoxic, biodegradable, and biocompatible; sodium alginate also contains polyelectrolytes that have amino and carboxyl groups, thus enabling the formation of microparticles via electrostatic attraction^27^. However, there are few reports about the application of poloxamer or sodium alginate in endoscopy. During the oesophageal ESD process, soon after mucosal resection, the colloidal solution is initially spread on the artificial ulcer, and the fixative solution is then sprayed on it. Calcium chloride can interact with sodium alginate quickly, resulting in molecular entanglements. Next, poloxamers transform into a semisolid gel at body temperature, leading to further protection. Covering post-ESD wounds with this gel protects the artificial ulcer against digestive juices and accelerates mucosal regeneration.

In this multicentre, randomized, single-blind trial, we investigated the effectiveness and safety of a novel gel designed to protect oesophageal wounds resulting from ESD.

## Methods

### Study design

This multicentre, randomized, single-blind trial performed in four hospitals in China investigated the efficacy and safety of a novel protective gel applied after oesophageal ESD. The study protocol was approved by the ethics committee of Zhejiang University School of Medicine First Affiliated Hospital (PRO20200140) and was executed in compliance with the Declaration of Helsinki, Good Clinical Practice guidelines, and applicable local regulations. This trial was registered on http://www.chictr.org.cn/index.aspx (27/11/2021, ChiCTR2100053692).

### Participants

The study included adults who underwent oesophageal ESD between December 2020 and November 2021 across four participating hospitals. The inclusion criteria were as follows: (1) age 18–80 years, (2) underwent oesophageal ESD successfully, (3) first resection of the same lesion and (4) written consent provided for study participation. The exclusion criteria were as follows: (1) diagnosis of active infection or severe systemic diseases, (2) diagnosis of coagulative dysfunction, hepatic or renal failure, or cardiopulmonary dysfunction, (3) diagnosis of other tumours, (4) a history of oesophageal surgery or radiotherapy, (5) inability to complete the ESD operation, (6) pregnancy, (7) glucocorticoid therapy in the past 6 weeks and (8) refusal to participate in the study.

### Randomization and masking

Using a random number table, participants were randomly divided into the control and experimental groups at a 1:1 ratio. Sequence generation, participant enrolment, and assignment were performed by three different individuals who were not involved in the rest of this clinical trial. In this single blind trial, masking of the allocations was only attempted for participants in this study.

### Procedures

In the control group, participants only underwent a standard oesophageal ESD procedure, whereas those from the experimental group underwent application of this new gel after oesophageal ESD. ESD was performed by experienced endoscopists at each participating institution. For the ESD procedure, an upper gastrointestinal endoscope (GIF-Q260J Endoscope, Olympus, Tokyo, Japan) and a dual knife (Olympus, Tokyo, Japan) were used. The lesion was marked using dots, created by a dual knife. The lesion was lifted by injection into the submucosal layer by a solution stained using indigo carmine, and then submucosal dissection was performed.

Hangzhou Yingjian Bioscience & Technology Co., Ltd provided the protective gel for free. This gel consists of colloidal and fixative solutions. The colloidal solution contains poloxamers, sodium alginate, and beta-glucan, whereas the fixative solution consists of calcium chloride. In the experimental group, after oesophageal ESD, the colloidal solution was first spread onto the ulcer, followed by the fixative solution.

**Figure 1:**
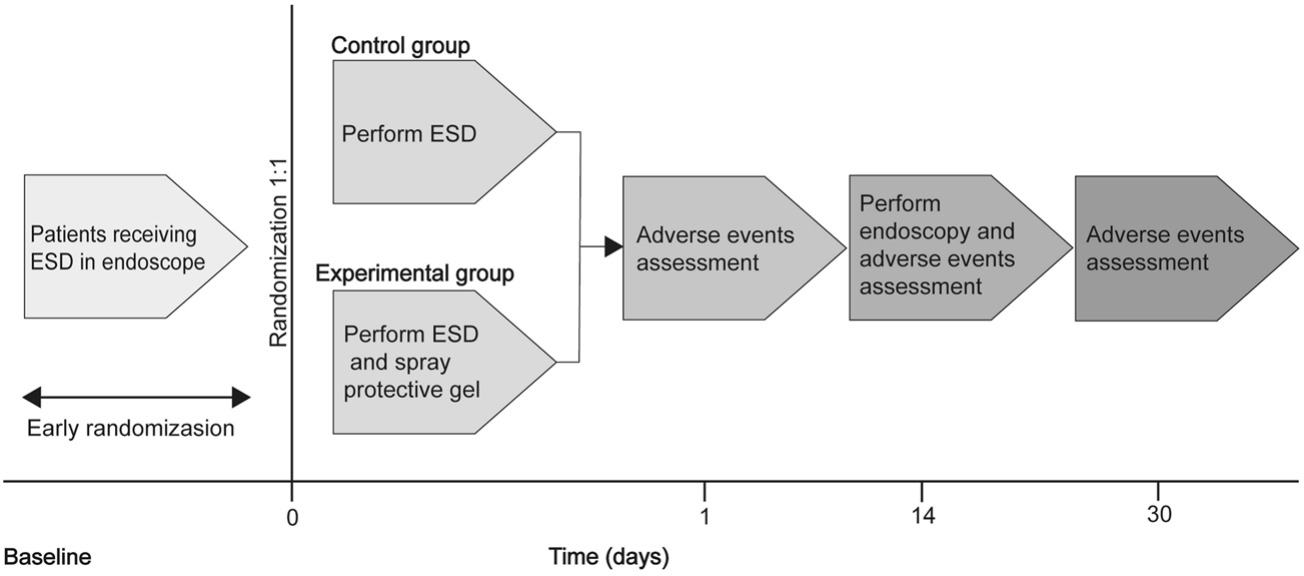
Study design. Participants were randomly divided into the control and experimental groups at a 1:1 ratio. In the control group, participants only underwent a standard oesophageal ESD procedure, whereas those from the experimental group underwent application of the gel after oesophageal ESD. A repeat endoscopy was performed, during the 2-week follow-up. Participants were instructed to report any discomfort on post-ESD days 1, 14, and 30. ESD, endoscopic submucosal dissection.

Following ESD, participants were instructed to report any discomfort, such as fever, chest pain, or dysphagia, on post-ESD days 1, 14, and 30. A repeat endoscopy was performed, during the 2-week follow-up, to confirm resection site healing, assess gel status, and detect any delayed bleeding, perforation, or dysphagia (Fig. 1).

### Outcomes

The primary outcome of this study was the healing rate. Secondary endpoints included ulcer area 14 days later, absolute change of ulcer area, and wound characteristics in endoscopic re-evaluation 14 days after ESD. The calculated assessment metrics were as follows:

1. *Ulcer area 14 days later* The ulcer area was measured using the same kind of reference through endoscopy using the ImageJ 1.53 analysis system (National Institutes of Health, Bethesda, MD, USA). To be short, the real ulcer area = (The area of wound detected from endoscopy)/(The area of reference detected from endoscopy) × (The real area of the reference) (Fig. S1).
2. The absolute change of ulcer area = (area of initial ulcer) − (ulcer area 14 days later). Area of initial ulcer was measured using the formula mentioned above (Fig.S1).
3. The healing rate = [(area of initial ulcer) − (ulcer area 14 days later)]/(area of initial ulcer) × 100%.

The primary and secondary endpoints were centrally assessed among the participants completing the whole process. Safety outcomes were adverse events, including fever, chest pain and dysphagia, which were analyzed among all the participants who were randomly assigned and completed oesophageal ESD. Adverse events were coded by a coding specialist, and were tabulated by system organ class and preferred terms using the MedDRA dictionary, version 19.0.

### Statistical analysis

The sample size was calculated based on the primary studies. The treatment difference assumption was mainly based on another study^28^. A sample size of 46 patients per randomized group was required to achieve 90% power in our statistical analysis and show statistical significance at the one-sided α level of 0.025 (assuming a true difference of 25% and a dropout rate of 10%). The primary and secondary outcomes were assessed in the population completing the whole process. Safety analyses were performed among all the patients who were randomly assigned and completed the oesophageal ESD.

Adverse events were organized by our study group and presented as the number and proportion of patients. For other surgical information, the surgical time was defined as the interval between the start and completion of oesophageal ESD. The cutting time was the interval between the start of the resection and complete separation of the specimen. Moreover, the protective gel injection time and the wound coverage by gel were also calculated among the experimental groups.

The results are presented as the mean ± standard deviation or counts (percentages). The t-test was used for data analysis. All *P* values in this trial were two-sided, and *P*—values < 0.05 were considered to be statistically significant. All statistical analyses were performed using SPSS software, version 25.0.0.0 for macOS, or Prism 8.4.0 for macOS.

### Ethics approval

All study procedures were approved by the ethics committee of Zhejiang University School of Medicine First Affiliated Hospital (PRO20200140). The authors have obtained informed consent from all subjects and/or their legal guardian(s).

## Results

### Study flow

**Figure 2:**
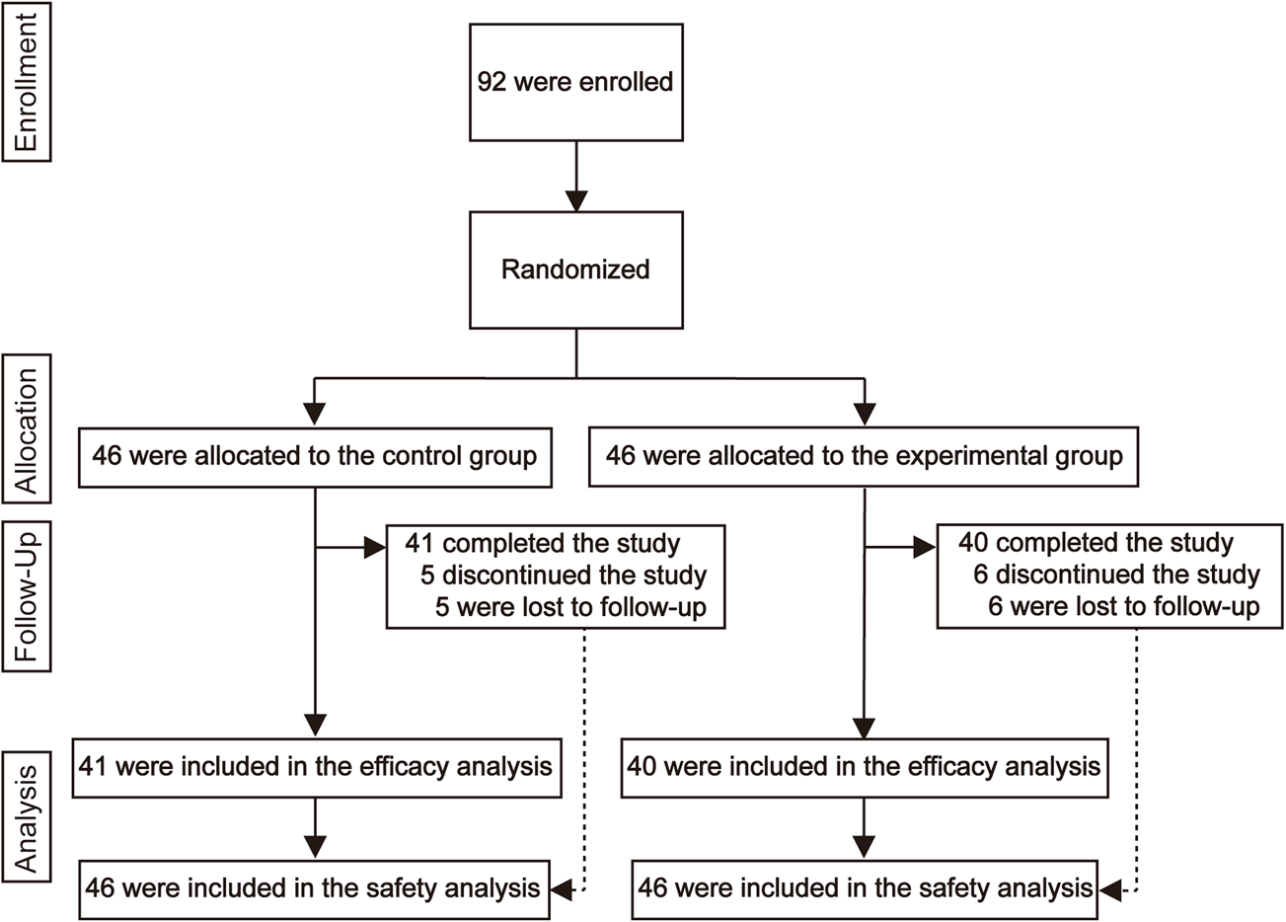
Trial profile. The primary outcome (the healing rate) and secondary outcomes (ulcer area 14 days later, absolute change of ulcer area, and the wound characteristics in endoscopic re-evaluation 14 days after ESD) were assessed in the population completing the whole process. The safety analyses were measured among all the patients who were randomly assigned and completed the oesophageal ESD. ESD, endoscopic submucosal dissection.

In this study, 92 patients were screened and considered eligible between December 2, 2020, and November 03, 2021. Among them, 46 individuals were randomly assigned to the control group, whereas the other 46 individuals were assigned to the experimental group. Overall, 81 (88.0%) of 92 patients completed the whole process and were included in the efficacy analysis, while the remaining 11 (12.0%) patients were lost to follow-up and discontinued the study. All 92 individuals were included in the safety analysis (Fig. 2).

### Baseline characteristics and wound characteristics

Baseline patient characteristics were similar between the groups (Table 1). Most patients in the control and experimental groups were male (33 [71.7%] and 32 [69.6%], respectively), and the mean ages of patients in these groups were 65.8 ± 8.1 years and 66.0 ± 7.2 years, respectively (Table 1). Generally, we observed no intergroup differences in mean age, height, weight, body mass index, or other baseline characteristics.

**Table 1:** Patients’ baseline characteristics.

Characteristic	Control group (n = 46)	Experimental group (n = 46)	*P* value
Sex, n (%)			0.819
Male	33 (71.7%)	32 (69.6%)	
Female	13 (28.3%)	14 (30.4%)	
Mean age (SD), y	65.8 (8.1)	66.0 (7.2)	0.672
Mean height (SD), cm	163.2 (6.6)	163.7 (6.3)	0.670
Mean weight (SD), kg	59.8 (10.5)	63.1 (8.6)	0.140
BMI (SD), kg/m^2^	22.5 (4.1)	23.6 (2.9)	0.173
Systolic blood pressure (SD), mmHg	128.7 (14.0)	132.3 (17.6)	0.274
Diastolic blood pressure (SD), mmHg	73.3 (9.1)	76.2 (9.9)	0.154
Temperature (SD), °C	36.7 (0.3)	36.7 (0.4)	0.977
Pulse (SD),/min	76.3 (10.8)	77.9 (12.2)	0.492
Respiration (SD),/min	18.0 (0.8)	18.2 (0.9)	0.703

There were no significant differences (*P* < 0.05) between the study groups. Data are n (%) or mean (SD).

*BMI* Body-mass index.

**Figure 3:**
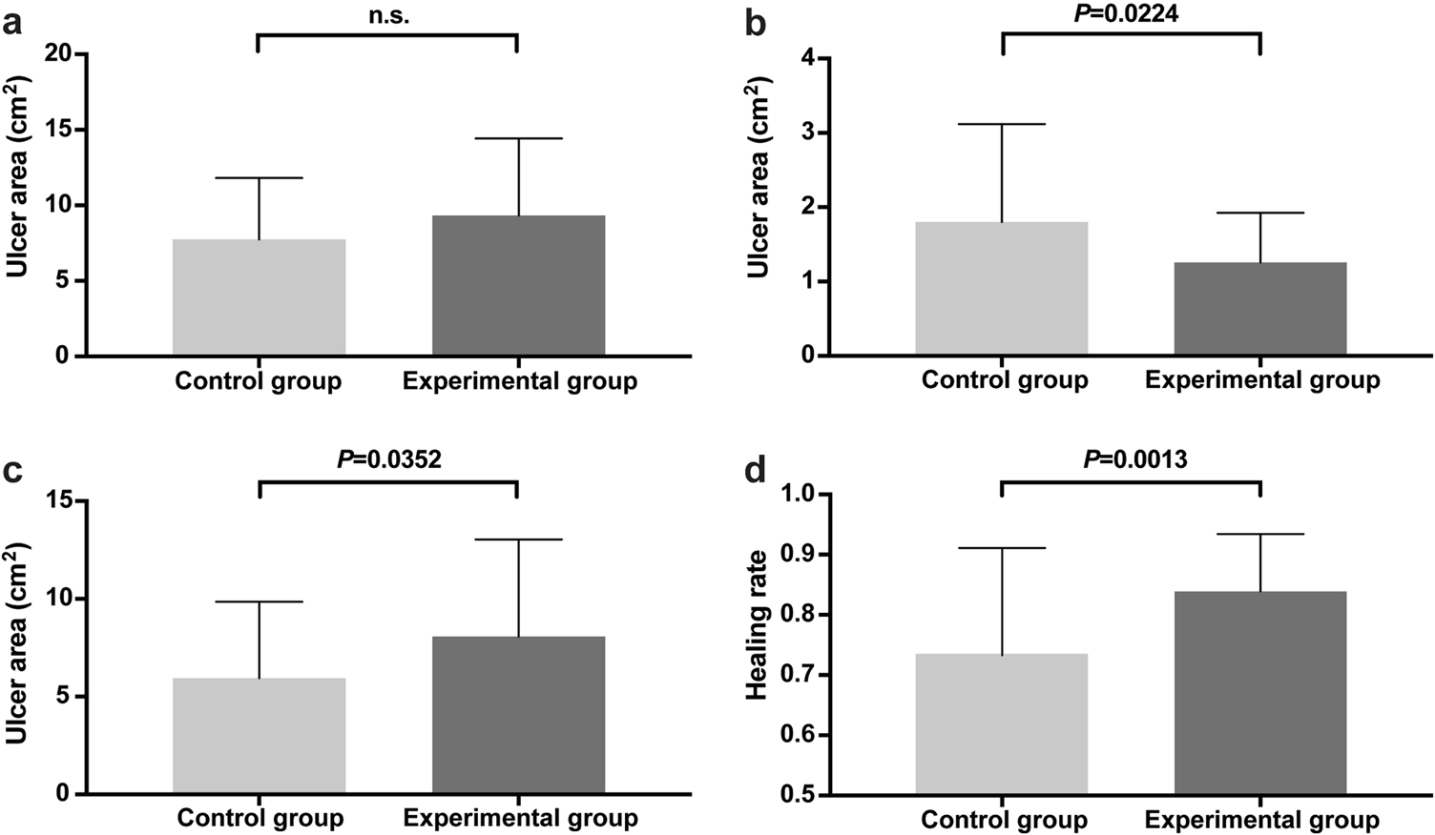
Calculated ulcer areas and the healing rate. (**a**) The initial ulcer area with no statistically difference between two groups. (**b**) The remaining ulcer area 14 days after ESD was much larger in the control group than in the experimental group. (**c**) The absolute change of ulcer area in the control group and experimental group were 5.95 ± 3.90 cm^2^ versus 8.07 ± 4.97 cm^2^ (*P* = 0.0352). (**d**) The calculated healing rates. The healing rates were significantly higher in the experimental group than in the control group (83.89 ± 9.51% vs. 73.28 ± 17.81%, respectively, *P* = 0.0013). Data are presented as the mean ± SD. ESD, endoscopic submucosal dissection.

Altogether, 92 individuals completed oesophageal ESD successfully, and the procedure time was similar between the two groups (53.0 ± 21.3 min vs. 52.9 ± 19.0 min, *P* = 0.984) (Table 2). Similarly, there was also no significant difference in cutting time between the control and experimental groups (35.1 ± 17.5 min vs. 33.4 ± 16.6 min, *P* = 0.638) (Table 2). In this study, this protective gel successfully covered approximately 91.1 ± 3.6% of the area of the artificial ulcer in 2.1 ± 2.7 min. These results indicate the convenience of protective gel application. The endoscopically measured initial ulcer area was approximately 7.7 ± 4.1 cm^2^ in the control group and 9.3 ± 5.1 cm^2^ in the experimental group without a significant difference (Fig. 3a).

**Table 2:** Details of the endoscopic submucosal dissection procedures.

	Control group (n = 46)	Experimental group (n = 46)	*P* value
Procedure time (SD), min	53.0 (21.3)	52.9 (19.0)	0.984
Injection time (SD), min	–	2.1 (2.7)	–
Cutting time (SD), min	35.1 (17.5)	33.4 (16.6)	0.638
Covering range (SD), %	–	91.1 (3.6)	–

Data are mean (SD) unless otherwise stated.

### Evaluation of ulcer healing

**Figure 4:**
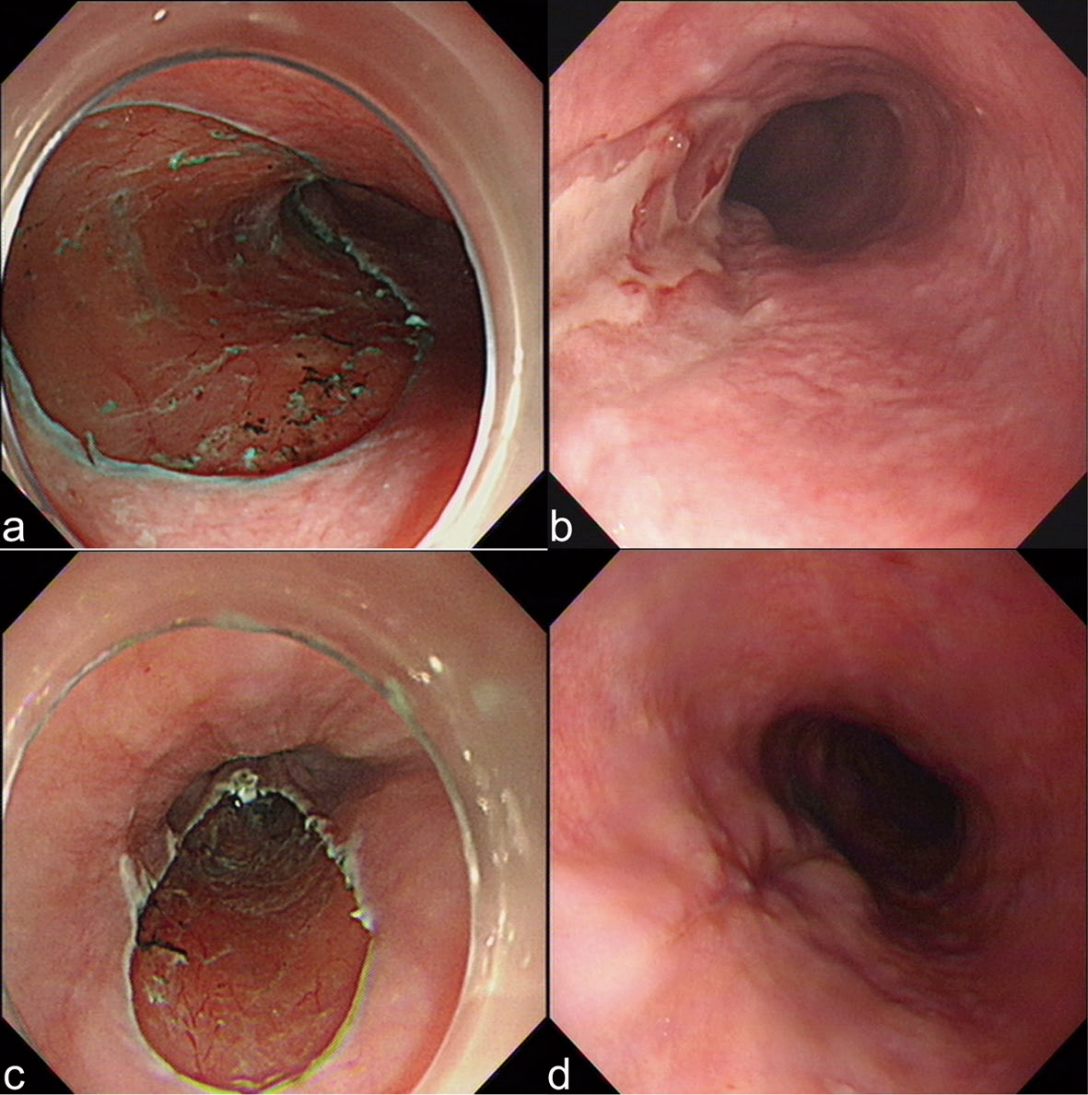
Artificial wounds detected from endoscopy. (**a**) Artificial ulcer remained from ESD in the control group. (**b**) The ulcer bed 2 weeks later in the control group. (**c**) The wound of ESD in the experimental group. (**d**) The ulcer bed 2 weeks later in the experimental group. The wounds in the experimental group showed less severity than the control group when detected by endoscopy 2 weeks later. ESD, endoscopic submucosal dissection.

Assessed from the endoscopic re-evaluation 2 weeks after oesophageal ESD, the remaining ulcer area was much larger in the control group than in the experimental group (1.80 ± 1.32 cm^2^ vs. 1.26 ± 0.67 cm^2^, *P* = 0.0224) (Fig. 3b). Additionally, the calculated absolute ulcer area changes and healing rates in the control group and experimental group were 5.95 ± 3.90 cm^2^ versus 8.07 ± 4.97 cm^2^ (*P* = 0.0352) (Fig. 3c) and 73.28 ± 17.81% versus 83.89 ± 9.51% (*P* = 0.0013) (Fig. 3d), which made it more convincing that this kind of protective gel could speed up ulcer healing. Furthermore, when detected by endoscopy 2 weeks later, wounds in the control group showed greater severity, accompanied by severe necrosis and oedema compared with the experimental group. We did not detect any residual gel over the ulcer bed in endoscopic re-evaluation (Fig. 4).

### Adverse events

On the first day after oesophageal ESD, 11 (23.9%) patients in the control group, and 19 (41.3%) patients in the experimental group, showed signs of adverse events, most of which were fever, chest pain, or abdominal pain (Table 3). However, these participants recovered without any additional medical treatment. Further evaluation indicated that these adverse events were related to the ESD procedure itself rather than protective gel application. There were no other adverse events such as delayed bleeding, delated perforation, or dysphagia reported in the 2-week and 1-month follow-ups (Table 3). These results indicate that this gel could be applied after ESD safely.

**Table 3:** Adverse events.

Time	Adverse events	Control group	Experimental group
1 day	Fever, n/N (%)	6/46 (13.0%)	12/46 (26.1%)
	Chest pain, n/N (%)	2/46 (4.3%)	5/46 (10.9%)
	Abdominal pain, n/N (%)	3/46 (6.5%)	0
	Dizziness, n/N (%)	0	1/46 (2.2%)
	Cough, n/N (%)	0	1/46 (2.2%)
14 days	Adverse events	0	0
30 days	Adverse events	0	0

Data are n/N (%). The adverse events of the day after endoscopy were measured among all the patients who were randomly assigned and completed the endoscopic submucosal dissection. And the follow-up 14 days later or 30 days later only measured the participants that continued the study.

## Discussion

In this multicentre, randomized, single-blind trial, we validated the potential of our novel protective gel as an effective and safe therapeutic agent for artificial wounds after oesophageal ESD. The healing rates of ulcers treated with this protective gel were much higher than those observed in the control group. Patients treated with the gel also had less severe wounds as determined by repeat endoscopy. Additionally, we observed no severe adverse events related to gel application that were reported during the 1-month follow-up. Therefore, our findings suggest that this kind of protective gel should be assessed as a potential treatment for patients receiving oesophageal ESD.

ESD has enabled the en bloc resection of large-sized superficial oesophageal neoplasms, but it carries the risk of causing oesophageal stricture, bleeding, and perforation^9^. Among them, stenosis is the main long-term complication of oesophageal ESD and can substantially impair the patient’s quality of life. Currently, there are many prophylactic options to prevent oesophageal stricture after ESD, including corticosteroid administration, polyglycolic acid (PGA) sheets, endoscopic balloon dilation (EBD), and autologous cell sheet transplantation^29^. However, this kind of protective gel that consists of colloidal and fixative solutions has never been reported in the current literature. Poloxamers have been used in tissue engineering, such as neurogenesis, angiogenesis, and bone regeneration. However, most of the research focused on wounds on the skin. The environment of the digestive tract is quite different from the skin surface. For example, there are some digestive juices and foods in the oesophagus that cannot be removed completely through peristalsis. By interacting with the wounds, these things may delay the healing process. Therefore, it is essential to assess the tissue repair ability of poloxamer in the digestive tract.

During the wound healing process, the infiltration of inflammatory cells, excessive collagen formation, and disorganized fibrosis might play a role in stricture formation^12^. Therefore, reducing the inflammatory reaction, promoting rapid re-epithelialization, and minimizing the damage will prevent oesophageal stricture after ESD^30^. In this study, the healing rate in the experimental group was significantly higher than that in the control group, which indicates that this kind of protective gel could strongly accelerate the re-epithelialization process after oesophageal ESD. The protective effects of the gel can be attributed to the following underlying mechanisms. First, this protective gel could cover the artificial ulcer and protect it from harmful microbiological factors as well as chemical factors in the gastrointestinal tract. Second, poloxamers have been demonstrated to encompass healing characteristics. Poloxamers could stimulate the expression of vascular endothelial growth factor (VEGF) as well as transforming growth factor-β (TGF-β), which could strengthen the wound healing process. They could enhance tissue granulation along with fibroblast proliferation^31^. Third, the emergence of bacterial biofilms in most chronic wounds could hinder wound healing since they can endure many antibiotic and antimicrobial treatments. It was reported that poloxamers could improve biofilm removal^32^. Lastly, it is known that poloxamers could increase the performance of matrix metalloproteinase 2 and 9 gelatinases, while simultaneously inhibiting matrix metalloproteinase-8 collagenase. It is expected to accelerate autolytic debridement by degrading the damaged collagen and protecting the untouched collagen^33^.

Currently, steroid administration and repeated endoscopic balloon dilatation are common interventions for preventing oesophageal strictures. However, it was reported that intralesional steroid injections could inhibit the healing process and cause oesophageal perforation and gastrointestinal bleeding. Systemic administration increases the risk of immunosuppression and osteoporosis^2^. Similarly, the effect of endoscopic balloon dilatation is temporary, which could decrease patients’ quality of life and increase medical costs. Repeated oesophageal dilatation has been reported to carry a risk of perforation (0.4–1.1% per procedure and up to 9% per patient)^6,34,35^. During the 1-month follow-up, we observed adverse events, such as fever, chest pain, or abdominal pain on the day after oesophageal ESD in 30 patients in our study. The symptoms of these patients were mild and these individuals recovered soon without additional medical treatment. Further analyses showed that these adverse events were more likely a result of oesophageal ESD itself rather than gel application. No delayed bleeding or delayed perforation was reported during the follow-up period. The results of our study demonstrated that this kind of protective gel could be safely applied to artificial ulcer protection after oesophageal ESD.

As mentioned above, advanced strategies such as cell sheets or mineral sheets have been proposed to protect wounds. However, some of them are time-consuming, while others require high technical expertise^22^. Tissue engineering and autologous tissue transplantation require high technical surgeon expertise with an unavoidable longer procedure time^22,23^. Additionally, it is quite difficult to fix the PGA sheets on the wound. In this research, the protective gel could be applied to cover more than 90% of the ulcer area within approximately three minutes. This result indicated that this gel could be sprayed on the wound conveniently, which means that it could overcome the shortcomings of tissue engineering and PGA sheets to some extent.

Admittedly, there are some limitations in this study. First, most of the former studies have indicated that postoperative stricture formed after 14 days^2,15,30,36^. We performed repeated endoscopy 14 days after oesophageal ESD and did not perform a third endoscopy at the 1-month follow-up. However, there are some patients who do not experience dysphagia until 1 month later or even much longer. Therefore, a long-term study is needed in future research. Second, currently, oral steroid administration is most widely used to prevent stricture after ESD. The risk of oesophageal stricture increases considerably when ESD exceeds three-quarters of the oesophageal circumference^4–6^. It is recommended that these high-risk patients use oral steroids^37^. However, most artificial ulcers included in this research did not exceed three-quarters of the oesophageal circumference. Therefore, we did not set oral steroid administration as the control group and mainly measured the healing rate in this research. Further research comparing this gel with steroid administration could be performed. Finally, 11 patients were lost to follow-up and discontinued the study. These factors may cause some bias in this randomized controlled trial.

## Conclusion

In conclusion, this multicentre, randomized, single-blind trial emphasizes the effectiveness, convenience, and safety of our novel protective gel when applied over wounds following oesophageal ESD. It could be applied to cover more than 90% of the ulcer area within approximately three minutes. More importantly, no severe relative adverse events were reported during the 1-month follow-up. Therefore, we recommend using this protective gel as a prophylactic intervention to promote ulcer healing and protect the ulcer bed after oesophageal ESD.

## Supplementary Information

Supplementary Information.

## Data Availability

All the related data is available on reasonable request. The data that support the findings of this study have been deposited in http://60.191.20.66/wy_sgcd, and the data is available from the corresponding author, Zhe Shen, on reasonable request.
