# Supplementary material for: A new protective gel to facilitate ulcer healing in artificial ulcers following oesophageal endoscopic submucosal dissection: a multicentre, randomized trial
Source: Sci Rep. 2023 Apr 26;13:6849. doi: 10.1038/s41598-023-33982-7 (PMC10133223; doi:10.1038/s41598-023-33982-7)
Supplement: Supplementary file 1 — Supplementary Information. [file 41598_2023_33982_MOESM1_ESM.docx]

**Supplementary materials**

**A new protective gel to facilitate ulcer healing in artificial ulcers following oesophageal endoscopic submucosal dissection: A multicentre, randomized trial**

Tianyu Zhou, Xinli Mao, Lei Xu, Haifeng Jin, Li Cen, Caijuan Dong, Linying Xin, Jiali Wu, Weimiao Lin, Bin Lv, Feng Ji, Chaohui Yu, and Zhe Shen

**Table of contents**

**1. Figure S1**

**
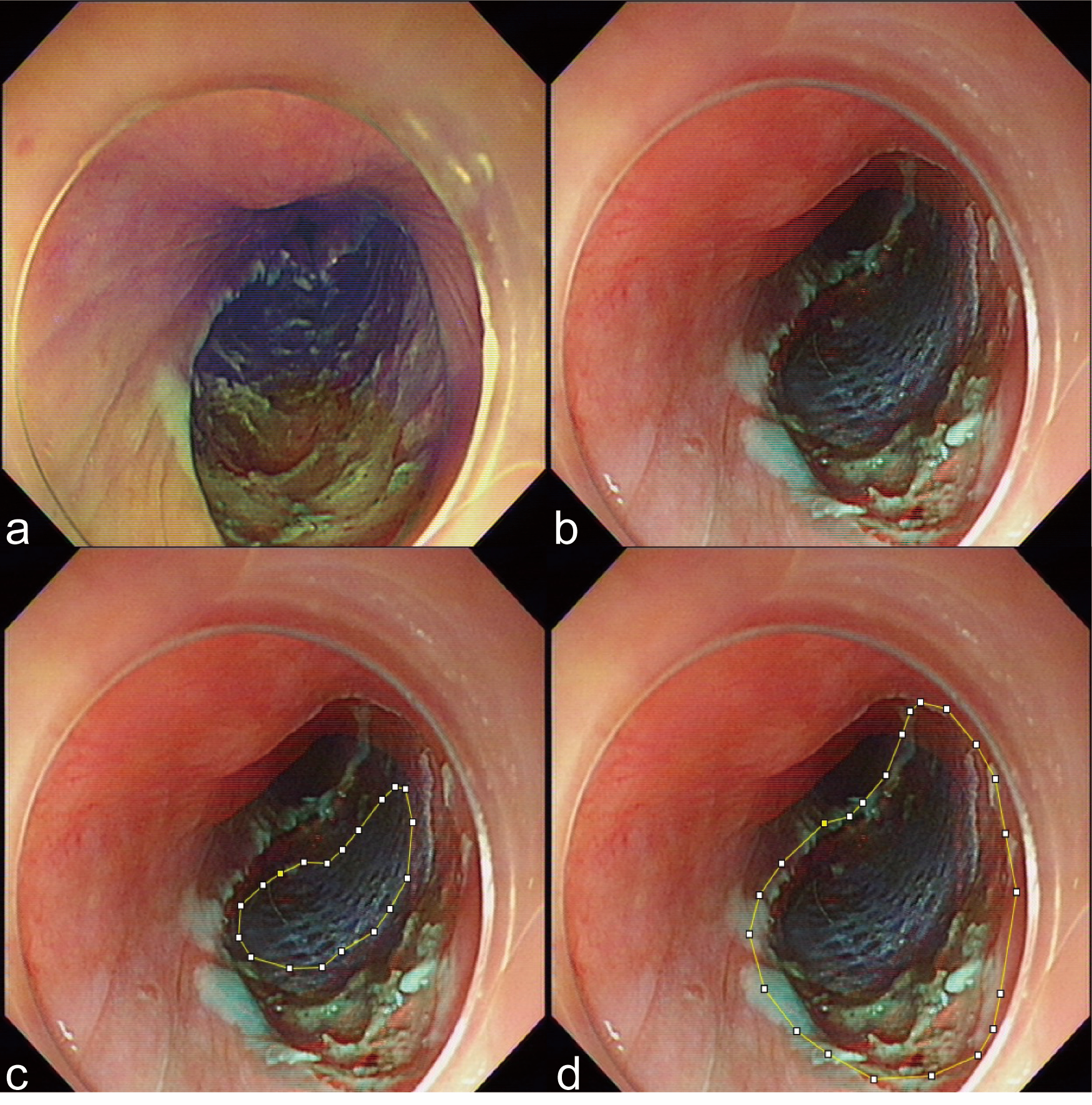
**

**Figure S1.** Protocol of measuring the ulcer area by ImageJ 1.53 analysis system. (a) The wound after oesophageal ESD. (b) Put the same kind of reference on the ulcer surface. (c) Measure the area of reference detected from endoscopy. (d) Measure the area of the wounds detected from endoscopy. The real ulcer area was then calculated using the following formula: (The area of wound detected from endoscopy) / (The area of reference detected from endoscopy) * (The real area of the reference).
